# Does optimal partitioning of color space account for universal color categorization?

**DOI:** 10.1371/journal.pone.0178083

**Published:** 2017-06-01

**Authors:** Yasmina Jraissati, Igor Douven

**Affiliations:** 1 Department of Philosophy, American University of Beirut, Beirut, Lebanon; 2 Sciences, Normes, Décision (CNRS), Paris-Sorbonne University, Paris, France; Universitat de Valencia, SPAIN

## Abstract

A 2007 study by Regier, Kay, and Khetarpal purports to show that universal categories emerge as a result of optimal partitioning of color space. Regier, Kay, and Khetarpal only consider color categorizations of up to six categories. However, in most industrialized societies eleven color categories are observed. This paper shows that when applied to the case of eleven categories, Regier, Kay, and Khetarpal’s optimality criterion yields unsatisfactory results. Applications of the criterion to the intermediate cases of seven, eight, nine, and ten color categories are also briefly considered and are shown to yield mixed results. We consider a number of possible explanations of the failure of the criterion in the case of eleven categories, and suggest that, as color categorizations get more complex, further criteria come to play a role, alongside Regier, Kay, and Khetarpal’s optimality criterion.

## 1 Introduction

Why do we draw the boundary between the English-language categories “blue” and “green” where we do? Is this boundary the same across languages? Following Boas [1] and Whorf [2], some have argued that color categories vary across cultures and languages [3–5]. In Zuni, for example, there is no term for “orange”; colors that speakers of English refer to as “orange” are included in the extension of the Zuni equivalent for “yellow” [6]. And in Berinmo, one category ranges across most of what we refer to as “green” and “blue” in English, while another ranges across part of what we call “green,” “yellow,” and “orange” in English [7, 8]. Berlin and Kay [9] published data on color lexicons of industrialized societies (e.g., Cantonese, Catalan, English, Japanese, Mandarin) and non-industrialized societies (e.g., Hausa, Hopi, Masai, Navaho, Papago, Tzeltal), gathered either directly by interviewing native speakers (as in the cases of Cantonese, Catalan, English, Japanese, Mandarin) and in situ (Tzeltal), or through existing ethnographic data (as in the cases of Hausa, Hopi, Masai, Navaho, Papago). Based on this data, Berlin and Kay argued that some color categories—the so-called basic color categories—were common to all languages. Specifically, they claimed that there were eleven color universals. The universal basic color categories are those lexicalized in English by “white,” “black,” “red,” “yellow,” “green,” “blue,” “brown,” “gray,” “orange,” “purple,” and “pink.”

But how can the Basic Color Terms Theory (BCTT) argue for the existence of color universals when there are languages that categorize the color domain as differently as do, for instance, Zuni, or Berinmo, and English? Berlin and Kay note that some exceptional languages do not fit this universal pattern; and in any case, such discrepancies do not imply that color categorization is linguistically relative for the BCTT. Indeed, the difference between color categorizations lies not in the natures of the categories identified, but in their number. Berlin and Kay claim that the color lexicon evolves. In any language featuring only two basic color categories, these categories are black and white. In any language featuring three basic color categories, the third category is red. More generally, in their view color universals are gradually encoded in the lexicon following a constrained evolutionary sequence: black, white, red, yellow, green, blue, brown, purple, orange, pink, and gray. (In this paper, color categories are referred to in small capitals, color sensations are in lowercase letters, and color terms are in lowercase letters in quotation marks.)

To forestall misunderstanding, we note that the term “universal” here refers to a set of eleven color categories that are *potentially* found across languages. In some languages, there are only two or three categories, in some languages there are five or six categories, in other languages still there are as many as eleven. Thus, all languages feature a subset of these eleven universals. In other words, though there are eleven universals, these universals are not observed across all languages at the same time. However, as lexicons evolve, a gradually increasing number of such categories are observed.

The BCTT hypothesizes both the universality of color categories and the evolution of color lexicons on the basis of some 98 languages. Today, the World Color Survey (WCS) offers color naming data for some 110 additional languages [10]. This data reveals some striking regularities in the way different languages partition color space, thereby confirming Berlin and Kay’s hypothesis, according to which languages featuring the same number of color categories partitioned the space in similar ways. In their 1969 monograph, relying mostly on the localization of focal points, Berlin and Kay had argued that languages featuring eleven categories should be expected to partition color space in similar ways, the same being true of languages featuring three, four, five categories and so on. This is the essence of their universality claim. The WCS data also confirms that some languages feature fewer basic color terms than others.

Several categorization models have been put forward that aim to explain at the same time the existence of universal tendencies in color categorization as well as the deviations from those tendencies [11–17]. Taking their cue from a suggestion by Jameson and D’Andrade [18], Regier, Kay and Khetarpal [19] have offered a particularly interesting model, which has in its turn inspired more research on categorization applied to color and other domains [20–24].

The suggestion by Jameson and D’Andrade was that irregularities in color space might interact with a cognitively motivated preference for informative naming systems, and that this interaction would explain the universal patterns in color naming as well as the occasional deviations from those patterns that the WCS reports. In their paper, Regier, Kay, and Khetarpal (RKK) formalize the hypothesized interaction of perceptual and cognitive factors and use computational simulations to show that, thus formalized, the interaction yields partitionings of color space that closely match how various natural languages categorize that space into the first three, four, five, and six categories of the BCTT’s evolutionary sequence—viz., white, black, red, yellow, green, and blue—while also showing that sometimes two or more rather different categorizations (for the same number of categories) can both (all) achieve optimal or near-optimal informativeness.

According to the BCTT, however, there are eleven color universals, not just six: almost all industrialized societies have terms for white, black, red, yellow, green, blue, but also for brown, pink, orange, purple, and gray. Such is the case of the twenty languages, most of which are from industrialized societies, reported in Berlin and Kay’s monograph [9] (see pp. 8–9, where the focal colors of the twenty studied languages are mapped.) These twenty languages range from languages as different as Lebanese Arabic (see a recent study for data revealing the expected partitioning in Saudi Arabic [25]), Hebrew, American English, Hungarian, and Japanese (see Stanlaw’s [26] study of the Japanese lexicon), to name only a few.

RKK make a number of methodological choices that will seem questionable to some (more about this in the general discussion). Nonetheless, it succeeds in accounting for the categorization of up to six color universals. In light of this important result, it is legitimate to ask whether the exact same formal model predicts as accurately the partitioning of the space into all of the eleven universals. RKK’s own paper reports no results concerning categorization going beyond six categories. In this study, we apply their model to the case of eleven categories and show that natural languages do not optimally partition color space into eleven categories, where optimality is understood in RKK’s sense. We conclude that factors besides the ones implemented in RKK’s model are likely to play a role in color categorization as well.

## 2 Optimality and the irregularity of color space

**Fig 1:**
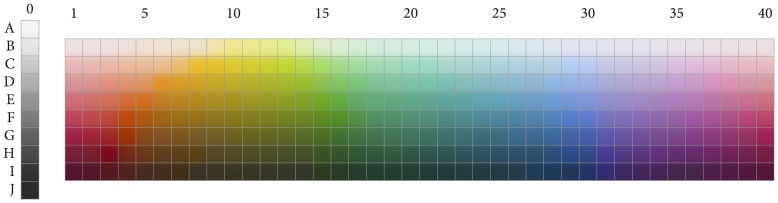
The 330 Munsell chips used as materials in the WCS.

**Fig 2:**
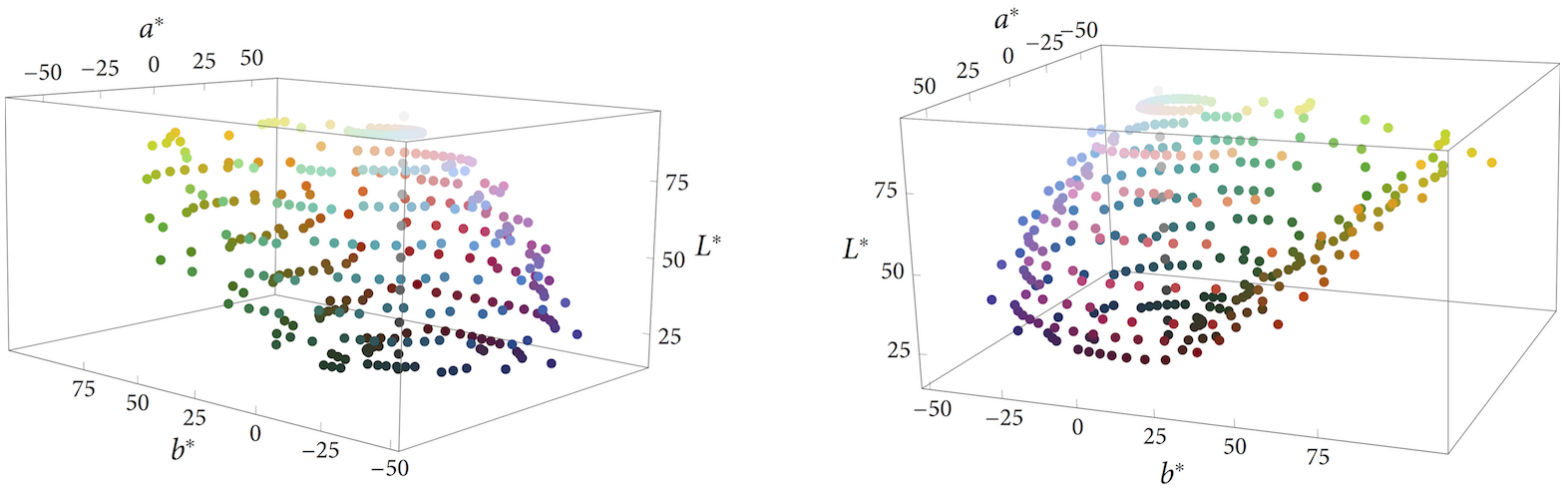
Different viewpoints of the set of 330 Munsell chips, placed in CIELAB space.

According to the proposal we are considering, color categorization results from an interaction of perceptual and cognitive factors. The perceptual factor is the irregularity of color space. Fig 1 shows the 330 color chips used for gathering the data reported in Berlin and Kay’s monograph [9] and in the WCS. These chips range across the surface of the Munsell system, a three-dimensional space that is generally taken to adequately represent human experience of color [27–29]. Jameson and D’Andrade [18] pointed to the irregularity—in the form of “bumps” and “depressions”—of the Munsell system. These bumps and depressions become particularly manifest when, as RKK did, one projects the 330 chips into CIELAB space; see Fig 2. Euclidean distances between pairs of points in CIELAB space have been found to measure fairly accurately perceived dissimilarities between the colors corresponding to the points (Fairchild, 2013).

Among other things, Fig 2 shows that the highly saturated yellow and red colors in the Munsell system form bumps at the surface of CIELAB space, while blue and green are not as saturated and correspondingly form depressions. The standard explanation of this difference between the levels of saturation reached is that our sensitivity thresholds are not uniform across the spectrum. Because the L- and M-photoreceptors in the retina are maximally sensitive to an overlapping range of wavelengths in the middle and long range, we are more sensitive in the red-green-yellow area than in the blue-green area, where the S-cones are sensitive to the short-wavelengths range, toward the blue end of the spectrum [30, 31].

The cognitive factor in RKK’s account of universal color categorization is a principle of optimal categorization. (It is similar to a principle put forward in Liljencrants and Lindblom’s [32] discussion of vowel spaces, as RKK point out; see the General Discussion.) According to this principle, a categorization is better, or more informative (as Jameson and D’Andrade would put it), the more similar the items *within* any of its categories are to each other and the more dissimilar they are to the items in the *other* categories [33, 34]. RKK operationalize the notion of optimal categorization by means of a well-formedness function *W*.

Let *P* be some partition of the Munsell chips used in the WCS. Then the function *S*,

$$S\left(P\right)=\sum_{x,y:\;P\left(x\right)=P\left(y\right)}\text{sim}\left(x,y\right),$$

measures the overall *similarity* between chips within the categories as determined by P, where the function sim(., .) is defined as exp(-0.001 × dist(., .) ^2^), with dist(., .) measuring CIELAB distance. (As the notation suggests, the summation in the definition of *S* is supposed to range over all the pairs of Munsell chips that according to *P* belong to the same category.) Furthermore, a function *D*,

$$D\left(P\right)=\sum_{x,y:\;P\left(x\right)\neq P\left(y\right)}\left(1-\text{sim}\left(x,y\right)\right).$$

measures the overall *dissimilarity* between chips belonging to what according to *P* are different categories. (Here, the summation ranges over all the pairs of Munsell chips that according to *P* belong to different categories.) RKK then define the well-formedness of color categorization *P* as

W(P)=S(P)+D(P).

Thus, a categorization is well-formed to the extent that it maximizes similarity within categories and minimizes similarity across categories.

To find a partitioning of the 330 WCS chips that maximizes *W* for some given number *n* of categories becomes computationally too difficult even for small values of n: While the number of ways to partition the set into categories is finite, this number for *n* = 3 is already so large that the best one can hope for is an algorithm that will give a good approximation of the global maximum in a reasonable amount of time.

RKK turn to computer simulations to create color space partitionings (with three, four, five, and six categories) that maximize the well-formedness function *W*. They show that such simulated partitionings closely resemble partitionings of color space by natural languages as documented in the WCS. The simulations for each of *n* ∈ {3,…,6} proceeded by first randomly splitting the 330 chips into *n* categories. Then one chip after the other was selected, in random order, and assigned to the category that resulted in the greatest overall increase in *W*. This process of reassignment was repeated until convergence was reached. And the whole process was repeated for 20 different initial random sortings of the 330 chips. In their paper, RKK report the best (understood in terms of *W*) of the 20 results, for each *n*.

RKK do not provide the computational details of their optimization procedure, but there are currently a number of off-the-shelf algorithms available that implement the idea of maximizing intra-cluster similarities and minimizing inter-cluster similarities. These algorithms are able to cluster even moderately large data sets in a split second. This holds true, for instance, for the Partitioning Around Medoids (PAM) clustering algorithm as implemented in R, Mathematica, MATLAB, various libraries for Python, and other software packages. While PAM or similar algorithms do not guarantee that the optimal clustering will be found—such a guarantee could only be had by going through all possible sortings of the 330 chips, which, as said, is unrealistic—these algorithms are generally believed to often yield good approximations to the global maximum (which does not have exactly the same definition for the different algorithms). Specifically, that PAM can get stuck in a local, but not global, optimum has become much less of a concern now that modern computers make it easy to use a great many different starting positions, which for many data sets is enough to make it unlikely that the global optimum is missed. All PAM results to be reported in this paper were obtained by using 2500 randomly chosen starting positions.

**Fig 3:**
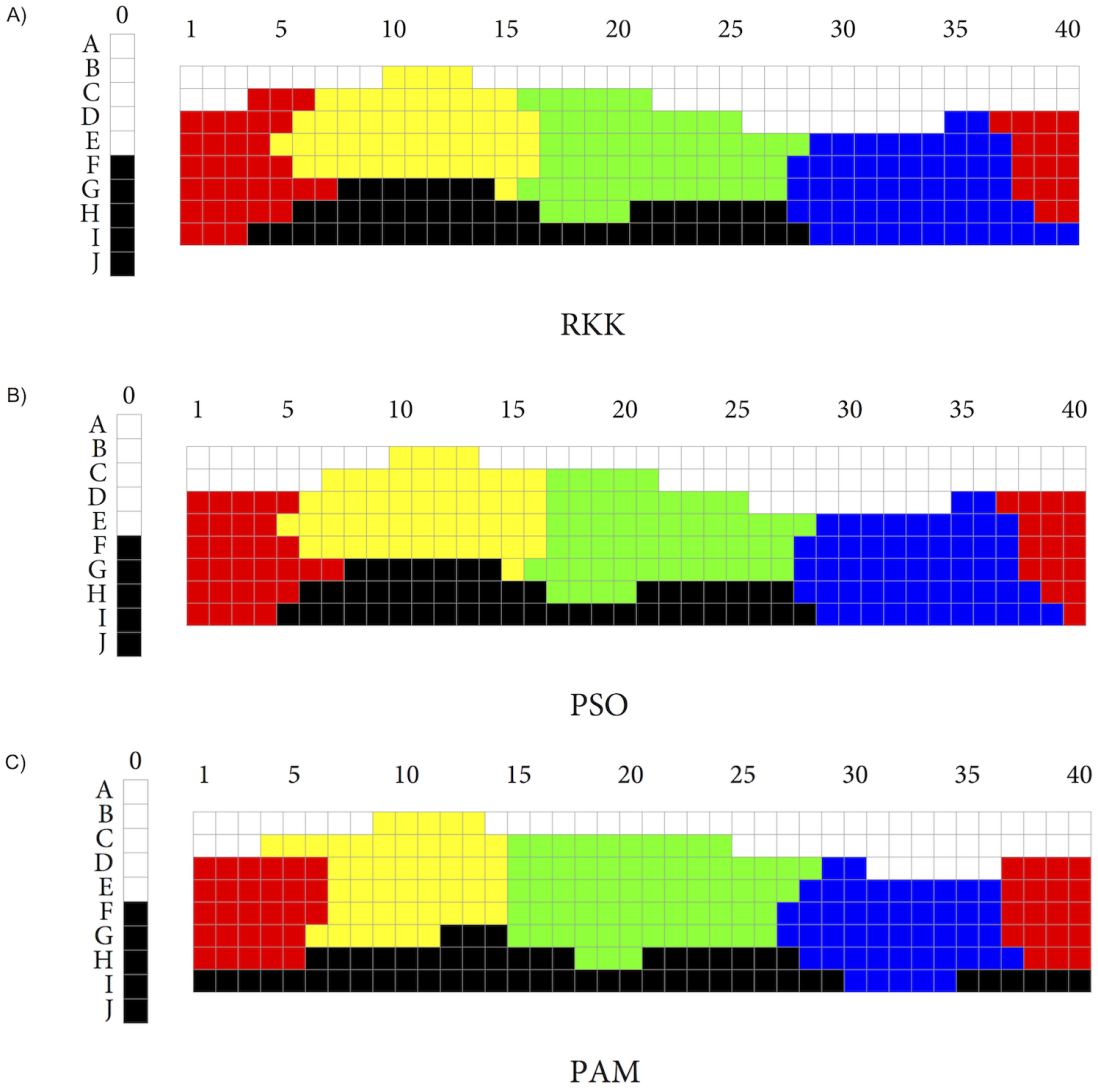
Solutions for n = 6.

**Fig 4:**
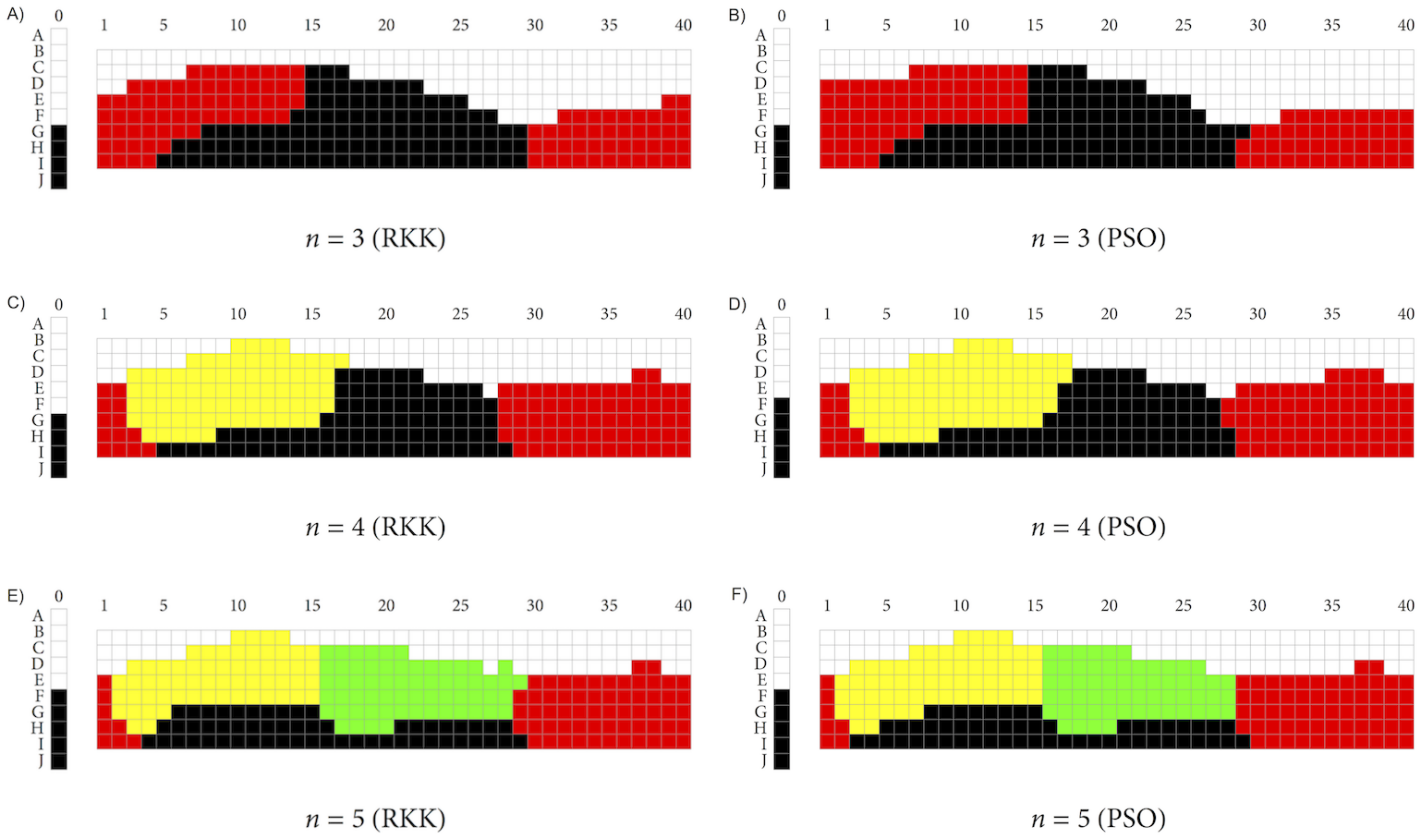
RKK and PSO solutions for *n* ∈ {3, 4, 5}.

We used the PAM function in the cluster package [35] for R to find the PAM solution for three, four, five, and six clusters of the 330 chips from the WCS, and we compared these with the solutions found by RKK. While PAM is not meant to maximize the well-formedness function W, the PAM solutions we obtained were visually close to the ones found by RKK, and the associated *W*-values of the former were also typically not much lower than those of the latter. However, we got solutions whose *W*-values were closer still to those of RKK’s corresponding solutions by using a function specifically custom-built for optimizing *W* via Particle Swarm Optimization (PSO; see [36]). This function made use of the hydroPSO package for R [37] and returned the best result of 100 randomly chosen starting partitions. The solutions obtained by this function were also visually very close to those obtained by RKK. Fig 3 shows RKK’s solution for *n* = 6 (Fig 3A) together with the solutions for the same number of clusters found by means of PSO (Fig 3B) and PAM (Fig 3C). Fig 4 shows RKK’s solutions for three (Fig 4A), four (Fig 4C), and five (Fig 4E) clusters side by side with the corresponding solutions found via PSO (respectively Fig 4B, 4D and 4F). And Table 1 compares the *W*-values for RKK’s solutions with those for the solutions found via PSO and PAM.

**Table 1:** *W*-values for RKK, PSO, and PAM clustering solutions.

*n*	RKK	PSO	PAM
3	39839.39	39717.24	39116.86
4	43845.73	43837.23	43702.00
5	45815.83	45804.50	45703.93
6	47062.66	47056.91	46969.94

The total CPU time for returning the best PSO solution for *n* ∈ {4, 5, 6} was between 45 and 50 minutes without parallelization and between 6 and 9 minutes with parallelization (on an Apple Macintosh, 3.2 GHz quad-core Intel Core i5 processor, and using the parallel package for R; for readers interested in running the optimization procedure themselves, the Appendix gives further details.) For later purposes, it is worth mentioning that for *n* = 3 we had to try several times to obtain a solution that was visually close to RKK’s. More often, we obtained a solution that had only a slightly lower *W*-value than the one reported in Table 1 but that appeared to show, next to categories for BLACK and WHITE, one for YELLOW-GREEN instead of RED.

From Figs 3 and 4, it is clear that PSO yields categories that largely overlap with those found by RKK; often, the categories differ on no more than one or two chips. The *W*-values of the PSO partitions are also exceedingly close to the *W*-values of the corresponding partitions found by RKK, with the possible exception of the *n* = 3 partition. Finding still better matches is only a matter of expending more computational resources. For the claims that are made in the discussion that follows, however, the approximations of the PSO solutions to RKK’s solutions are good enough.

## 3 Going beyond six categories

**Fig 5:**
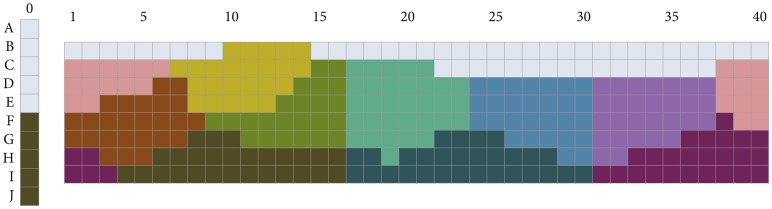
PSO solution for n = 11.

RKK’s simulations stop at *n* = 6, and one wonders what role *W* plays in finer-grained partitionings of color space. To investigate this question, we used the aforementioned custom-built function for optimizing *W* to obtain partitionings with more categories. Because there are eleven basic color terms in most languages of industrialized societies, we were in particular interested in what optimizing *W* would yield for that number of categories. The result for *n* = 11 is shown in Fig 5. In this figure, categories are colored according to the convention that a category *C* gets the color of the chip that, among the 330 chips in Fig 1, minimizes the Euclidean distance (in CIELAB space) to the centroid of the chips in *C*, where the centroid is the point in CIELAB space that minimizes the sum of the Euclidean distances to the points representing the colors of the chips in that category. (The L*-, a*-, and b*-coordinates of the centroid of category *C* are given by, respectively, the average of the L*-coordinates, the average of the a*-coordinates, and the average of the b*-coordinates of the chips in *C*. Note that these coordinates may not correspond to the color coordinates of any chip in the category.)

**Fig 6:**
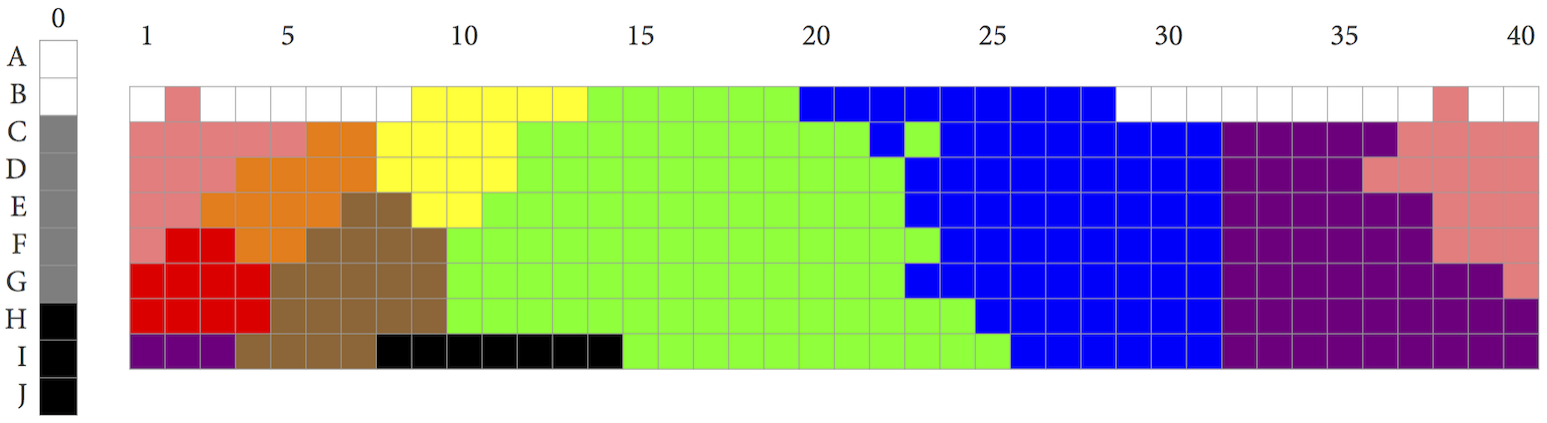
Mode map for French, from Claidière, Jraissati, and Chevallier (2008).

As explained above, both the BCCT and the WCS rest on a universality claim according to which languages featuring the same number of categories partition the space in similar ways. Given that French was reported as featuring eleven basic color terms [38], based on the universality claim, it is therefore expected to partition the color space in the same way as English, Arabic, Hungarian, Japanese, and so on. Thus, given the universality assumption, we compared the results of the *n* = 11 PSO solution with the French color naming data of Claidière, Jraissati, and Chevallier [39]. In their studies, Claidière and colleagues identified basic color terms exclusively via the criterion of inter-subjective consensus, a method that led them to include categories labeled “bordeaux” (“burgundy”) and “saumon” (“salmon”). For the purpose of our present study, in order to operate within the BCTT framework, we first reprocessed the raw data of Claidière et al.’s color naming studies, and then identified basic color terms using the BCTT criteria ([9], p. 5), leading to the exclusion of “saumon” and “bordeaux.” Indeed, “bordeaux” is the name of an object that is characteristically colored (i.e., the color of red burgundy wine), as is “saumon” (i.e., the color of the flesh of salmon fish), and both these terms can be considered as hyponyms, thereby violating two of the basic color terms criteria (viz., criteria ii and vi). The resulting partitioning, shown in Fig 6, is very similar to the mode maps of languages of other industrialized societies reported in Berlin and Kay’s monograph [9]. (The raw data is available at this link: Claidière, Nicolas, Yasmina Jraissati, and Coralie Chevallier. 2017. “French Color Naming Raw Data 2008.” Open Science Framework. May 14. osf.io/u5jf8.).

As can be seen from comparing Figs 5 and 6, there are striking differences between the partitioning for *n* = 11 obtained by PSO, and the universal partitioning as observed in the case of French. Looking at Fig 5 starting from the left, there is notably no category for RED, and the area included in the French “rouge” is divided between a dark PURPLE category and an ORANGE category. In addition, the PSO ORANGE category extends into much darker areas than does the French category labelled “orange,” PSO YELLOW extends much more toward the reds than does the French category labelled “jaune,” and more toward the greens into the French category labelled “vert.” Also, PSO BROWN is much wider than the French “marron,” and includes colors that are included in the French category for GREEN, “vert.” As a result, the PSO category for GREEN is much smaller than the French category “vert,” and includes only colors of medium brightness, and a few brighter colors. Most remarkably, there is a category in the PSO partitioning that does not appear in the French lexicon, a cyan type of color, lying between the extensions of PSO GREEN and BLUE, at medium and high values. Also, a category for dark turquoise, or TEAL, appears in the PSO partitioning, extending from under the CYAN category, into the BLUE, at low value. There is no such category in French. In French, the colors included in both these PSO categories are either in “vert” or in “bleu.” Finally, instead of the two French categories ranging across the purple area of the Munsell array—“violet” at the BLUE boundary, ranging across low and high values, and “rose” at the boundary of “violet” in areas of high value—in the PSO partitioning there are three categories. Apart from a PURPLE and PINK category, there is a DARK PURPLE category, mostly including colors that are included in the French “violet.”

There are a number of different ways to go beyond a purely visual comparison of the French naming data and the PSO result for *n* = 11. The first examines the various indices for comparing a clustering solution with a target clustering that statisticians have developed. Each in their own way, these indices purport to quantify the notion of closeness or similarity between a clustering and the target. There is considerable discussion about which of these indices is best, or even whether there is a unique best one. We therefore considered all of the most popular indices, hoping for unanimity in their qualitative verdicts.

Specifically, we considered the six most current indices for the kind of case at hand, to wit, the Adjusted Rand Index (ARI; [40]), the Split/Join distance (SJ; [41]), the Normalized Mutual Information index (NMI; [42]), the Variation of Information metric (VI; [43]), the Jaccard index (J; [44]), and the Fowlkes–Mallows index (FM; [45]). For present purposes, it suffices to know that the ARI has a range from -1 (complete disagreement between the clusterings) to 1 (complete agreement), yielding 0 when the agreement found equals the agreement one would expect to find by chance alone. The SJ distance measures the number of changes that have to be made to the one clustering to turn it into the other clustering, yielding a range from 0 (for identical clusterings) to two times the number of items in the domain. The fundamental notion underlying the NMI and VI indices is the reduction of uncertainty about whether a given item belongs to a given cluster in one clustering by knowing to which cluster it belongs in the other clustering. The indices use this notion somewhat differently. As a result, the NMI index has a minimum of 0 and a maximum of 1 (for identical clusterings), while the VI index also has its minimum at 0—which is now, however, reached for identical clusterings—and, with *n* the number of clusters, its maximum at 2log*n*, which for the relevant cases of *n* = 6 and *n* = 11 amounts to 3.6 and 4.8, respectively. (Thus, note that for the NMI index, higher is better, while for the VI index, lower is better.) The J and FM indices both have a maximum of 1, for identical clusterings, and a minimum of 0.

A comparison of the *n* = 11 partitioning obtained by maximizing RKK’s *W* function with the mode map for French shown in Fig 6 yields the following outcomes: NMI = 0.57; VI = 1.89; SJ = 269; ARI = 0.30; J = 0.24; and FM = 0.40. These results are clearly not good, in that each value is quite distant from the “optimal” point on the relevant scale. Admittedly, however, when it comes to interpreting the outcomes of these indices, there are no rules of thumb comparable to, for instance, the rules we have for interpreting the effect size of a t-test or an ANOVA.

A first way to get a clearer picture of what the aforementioned values tell us about the *n* = 11 partitioning is to compare them to the results for RKK’s own *n* = 6 case, which we can compare with the mode maps for the WCS languages with six color terms. Table 2 gives the results for each of these fifteen languages separately. From a glance at the results, it would appear that the *n* = 6 case is better than the *n* = 11 case: for indices for which high is better, almost all of the fifteen comparisons reported in Table 2 yield higher values than the values for those indices in the *n* = 11 case, and for indices for which low is better, the opposite holds.

**Table 2:** Popular cluster comparison indices for the *n* = 6 case for fifteen languages of the WCS.

Language (country)	NMI	VI	SJ	ARI	J	FM
Abidji (CI)	0.42	2.02	276	0.32	0.28	0.44
Buglere (PA)	0.58	1.48	188	0.45	0.38	0.55
Cavineña (BO)	0.48	1.67	227	0.27	0.27	0.44
Cofán (EC)	0.59	1.40	174	0.45	0.39	0.56
Guaymí (PA)	0.57	1.51	202	0.46	0.38	0.56
Kalam (PG)	0.53	1.65	204	0.45	0.38	0.55
Karajá (BR)	0.35	1.98	261	0.17	0.22	0.39
Martu-Wangka (AU)	0.56	1.48	212	0.42	0.36	0.54
Mixtec (MX)	0.54	1.57	203	0.37	0.32	0.49
Ocaina (PE)	0.59	1.40	182	0.42	0.36	0.53
Tabla (ID)	0.57	1.45	181	0.42	0.37	0.54
Tarahumara (MX)	0.46	1.76	247	0.27	0.27	0.44
Tboli (PH)	0.54	1.60	209	0.42	0.36	0.53
Vagla (GH)	0.56	1.46	215	0.40	0.35	0.53
Vasavi (IN)	0.57	1.52	174	0.47	0.40	0.57

*Note*. Country names are given in ISO codes.

However, a direct comparison of the two sets of results is complicated by the fact that cluster indices can be sensitive to the number of clusters. A better way to proceed, therefore, is first to compare both computational results at issue (RKK’s *n* = 6 case and our *n* = 11 case) with an appropriate baseline, and then to compare the results with each other by seeing how well they do with respect to their own baseline. More precisely, what we propose to do is to compare the values of the indices for the comparison of our *n* = 11 partitioning with the mode map for French with the results we obtain from comparing that same partitioning with randomly generated clusterings with eleven clusters, and to compare the results in Table 2 with the results we obtain from comparing RKK’s *n* = 6 case with randomly generated clusterings with six clusters, and then to see how much better than chance (if at all) the said cases do.

To establish a baseline for each of *n* = 6 and *n* = 11, we generated 1,000 random clusterings of the 330 chips shown in Fig 1, where each clustering was formed by first randomly choosing *n* points in CIELAB space and then assigning each chip to whichever of those *n* points its color was closest to (given its CIELAB coordinates), thus obtaining *n* clusters. The 1,000 random clusterings with six clusters were then compared with RKK’s *n* = 6 solution, calculating all of our six indices for each comparison, and the same was done for the 1,000 random clusterings with eleven clusters and our *n* = 11 solution. Table 3 shows how many of the random clusterings did better on a given index than the results from the comparisons above (for the *n* = 6 case, we have compared the outcomes of the simulations with the column averages of Table 2).

**Table 3:** Number of random clusterings (out of 1,000) that, according to the various indices, are better represented by the computational results than the relevant languages (the WCS languages with six color terms for RKK’s*nn* = 6 case, and French with eleven color terms for the *n* = 11 case).

	NMI	VI	SJ	ARI	J	FM
*n* = 6	148	190	104	199	166	188
*n* = 11	488	447	472	750	734	731

These results indicate that on average RKK’s *n* = 6 solution better captures the languages from the WCS with six color terms than it does the great majority of random clusterings with six clusters, and that this is so across all indices. Conversely, according to three indices, there is a close to 50 percent chance that a random clustering with eleven clusters is better represented by the *W*-optimal solution for *n* = 11 than the mode map of French with eleven color terms, and according to the remaining three indices, that chance is even around 75 percent. This means that for any random clustering with eleven clusters, it is as likely as not that the *W-*optimal *n* = 11 solution is a better representation of that clustering than of what we would like the solution to represent, to wit, the French lexicalization of color space.

Another indication that RKK’s procedure is much less suited for predicting the French color naming system than it is for predicting color naming systems with six categories comes from comparing both the *W*-value of the French mode map with those of the 1,000 random clusterings with eleven clusters and the W-values of the fifteen WCS languages with six color categories with those of the 1,000 random clusterings with six clusters. It turns out that the French mode map has a *lower W*-value than 841 of the 1,000 random clusterings with eleven clusters, while on average the languages with six categories have a *higher W*-value than 653.6 (SD = 288.9) of the 1,000 random clusterings with six clusters.

**Fig 7:**
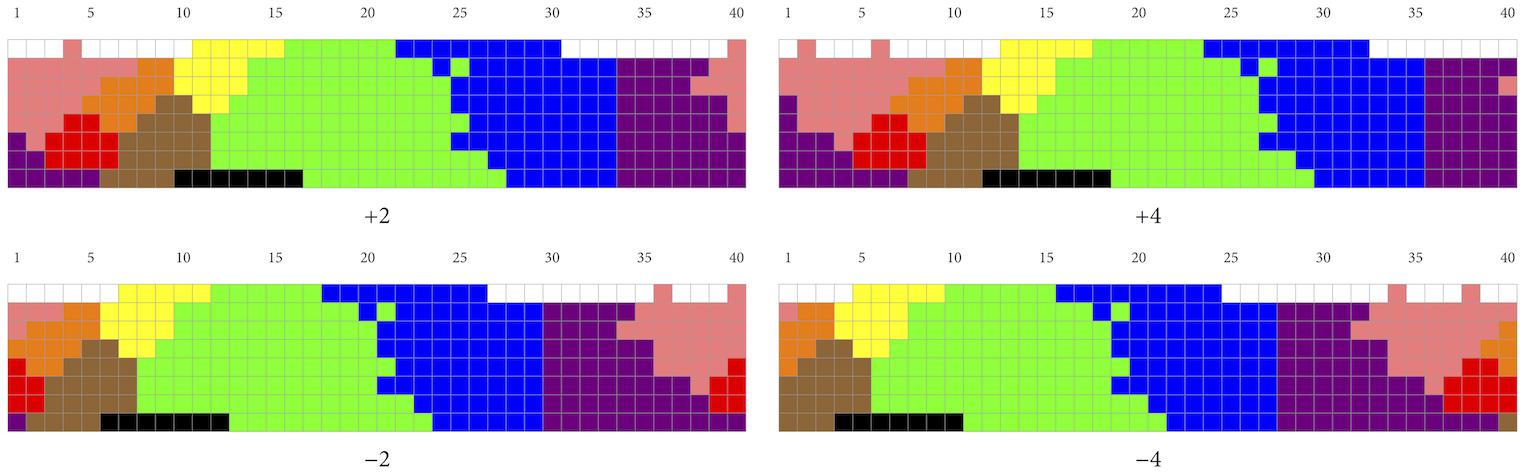
Four rotations of the mode map for French shown in Fig 6.

RKK themselves compare the *W*-values of the color naming systems of the WCS languages not with the *W*-values of random clusterings but with those of so-called rotations, where a rotation of a naming system “shifts” that system one or more columns, either to the left or to the right, along the hue dimension of Fig 1 (the 10 achromatic chips play no role in rotations), along which color lexicons mostly vary [21]. Fig 7 illustrates the concept by showing four rotations of the mode map of French.

Given that there are 40 hue columns, there are 40 possible rotations for each naming system (with the “0 column rotation” being the original naming system). As RKK point out, their hypothesis that color naming tends to reflect the actual structure of color space in conjunction with a preference for informative naming systems predicts that, in general, the original (unrotated) naming system has a higher *W*-value than any of its (non-trivial) rotations. This prediction is borne out by the data of RKK: the great majority of languages they examine maximize *W* when unrotated.

**Fig 8:**
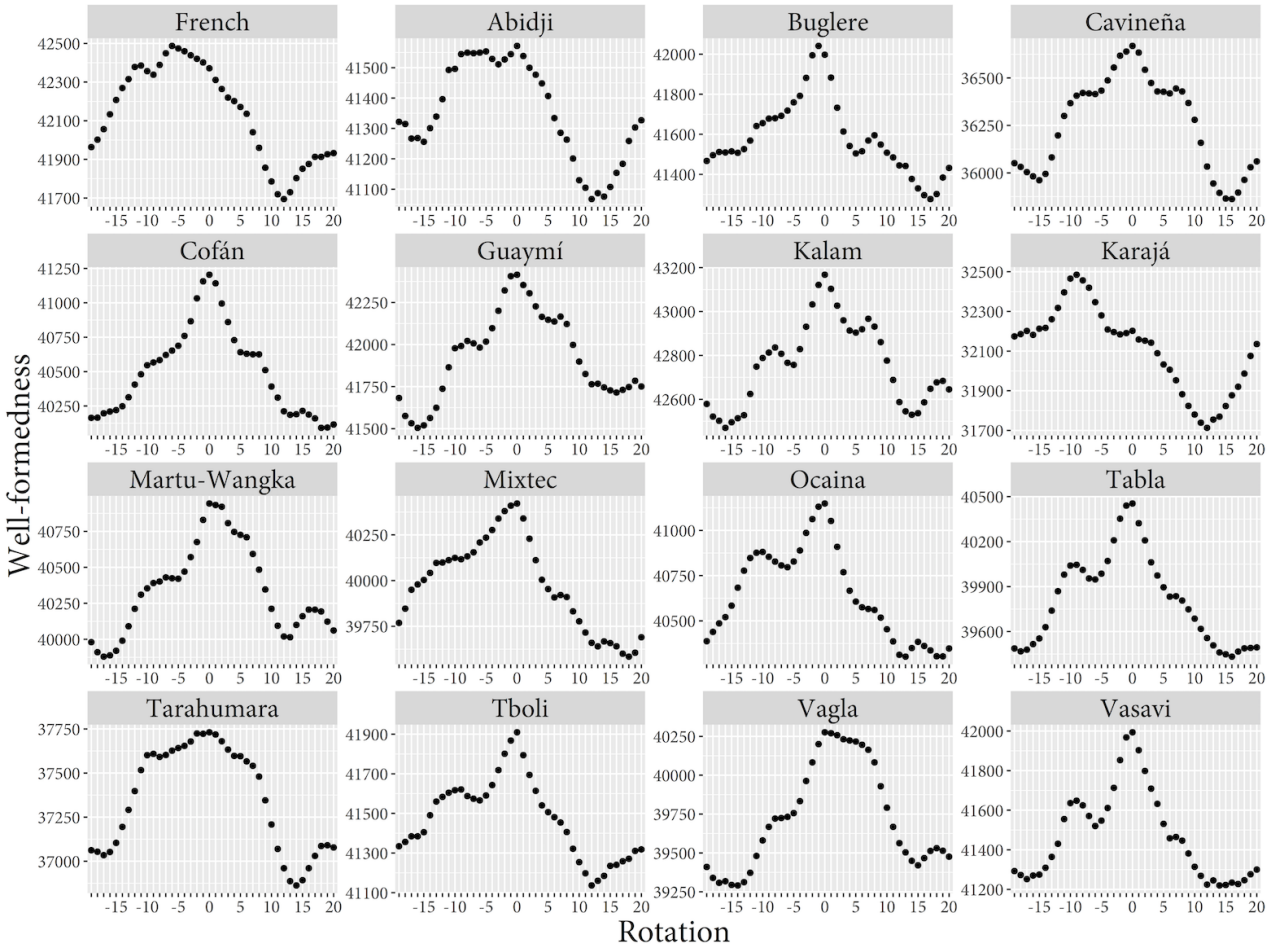
W values for the 40 rotations of French and the 15 six-category languages from the WCS.

Thus, rotations offer another way to compare how well optimality (as spelled out in terms of *W*) predicts the partitioning of color space into eleven categories—such as in the case of French—with how well optimality predicts the partitioning of color space into six categories, such as in the languages of the WCS with six color terms. For the French mode map as well as for the *n* = 6 mode maps, we calculated *W*-values for all their 40 rotations. The results are shown in Fig 8. Thirteen of the fifteen languages with six color terms reach maximum *W*-value when unrotated; one (Buglere) has its maximum immediately next to the 0 column rotation. For French, however, the picture looks very differently: no less than 10 rotations have a higher *W*-value than the original mode map. This is further evidence that RKK’s hypothesis works well for the *n* = 6 case but fails to generalize to the *n* = 11 case.

Our primary interest is the *n* = 11 case because there are alleged to be eleven color universals, and RKK’s categorization model succeeds in predicting the partitioning of color space into 3, 4, 5, and 6 categories. Nonetheless, it is worth looking briefly at the cases lying in-between the *n* = 6 case, which was still considered by RKK, and the *n* = 11 case. Do the results get worse gradually, for increasing number of categories, or might the *n* = 11 case stand out?

**Fig 9:**
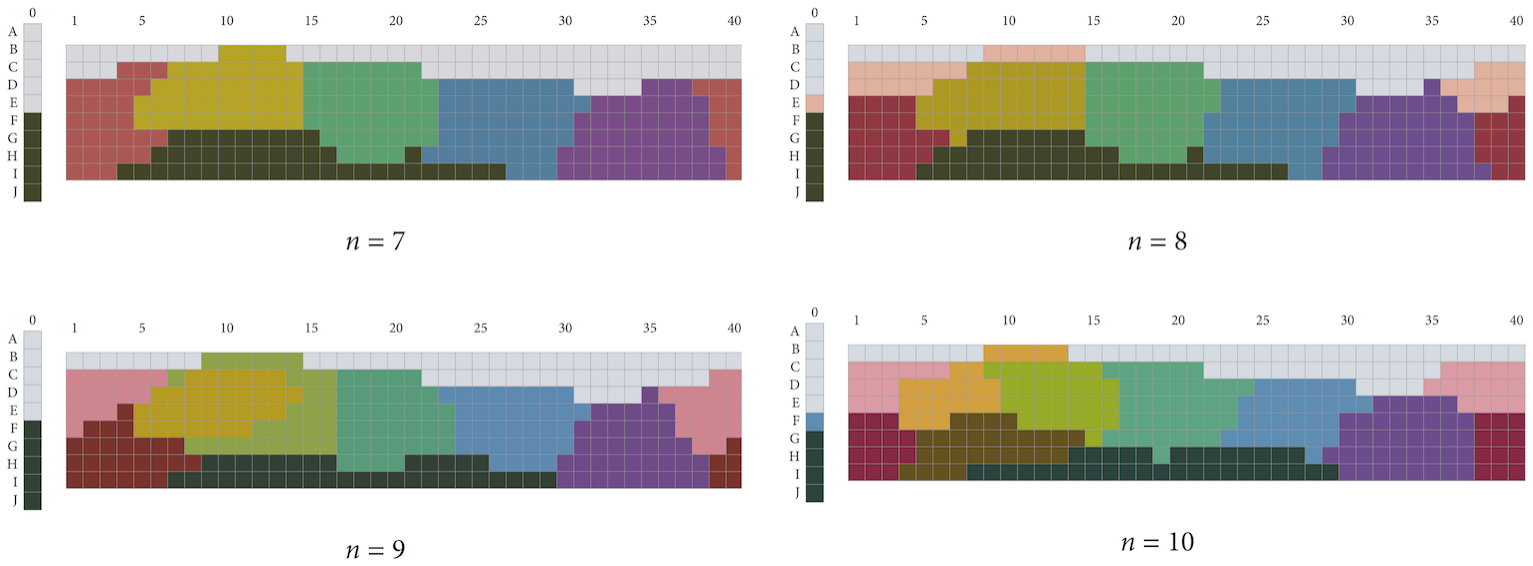
PSO solutions for n ∈ {7, …, 10}.

Fig 9 shows the PSO solutions for the intermediate cases, using the same coloring convention as was used for Fig 5. We see that, in the *n* = 7 case, there are two categories, blue and purple, dividing the blue–purple Munsell hue range, instead of one category, as in the *n* = 6 case. A PINK category appears when we move *n* = 8. It is difficult to tell by visual comparison whether these partitionings are strikingly different from those lexicalized by natural languages, because few WCS languages feature 7 or 8 color terms. Yet, in the cases of *n* = 9 and *n* = 10, the critical difference between the PSO solutions obtained and natural languages is that the BRIGHT GREEN category extending between GREEN and YELLOW at middle and high values in these PSO solutions is not reported in natural languages [14, 46]. BROWN only appears in the *n* = 10 solution.

**Fig 10:**
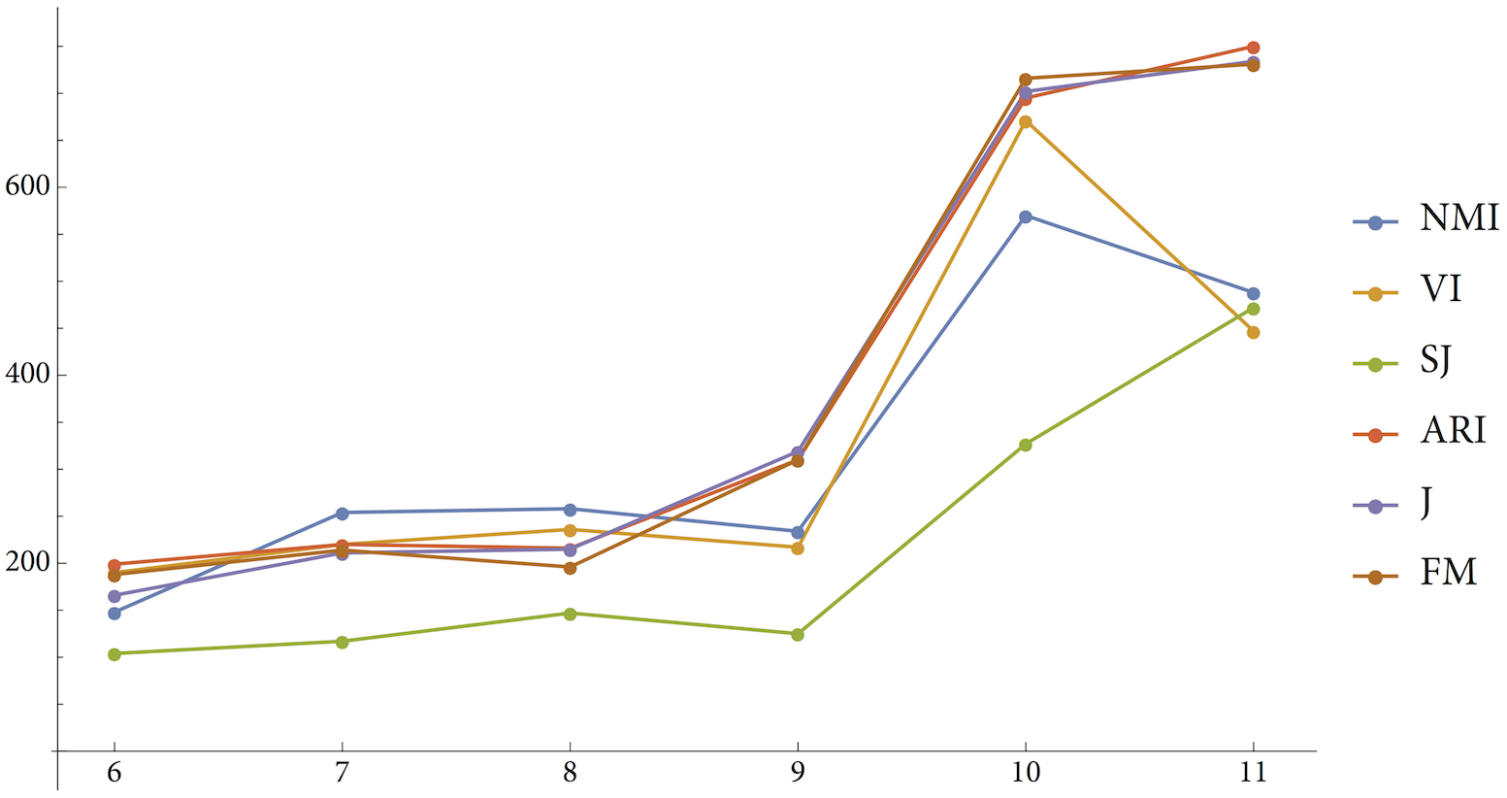
Number of random n-category clusterings that are better represented by the PSO solution for n categories than are the WCS languages with n color terms.

As we did for the *n* = 6 and *n* = 11 cases, we determined, for each of the PSO solutions shown in Fig 9, how many of 1,000 random clusterings were better represented (according to the earlier-used indices) by that solution than were the WCS languages with the corresponding number of color terms. Fig 10 gives a graphic summary of the results, including the *n* = 6 and *n* = 11 cases. The pattern in these data is impossible to miss. There is a clear increase in the number of “better represented” random clusterings over the series; Page’s test [47] shows this increase to be significant (*L* = 53468, *p* < .001). On the other hand, we also see that little changes when we go from six to nine categories, through the intermediate number of categories. The big jump comes when we go from nine to ten categories, with again little further change if we move on to eleven categories.

These comparisons do not by themselves shed a new light on why what worked so well in the *n* = 6 case—optimizing the function *W* on the 330 Munsell chips, taking into consideration the CIELAB distances between the colors of those chips—fails in the *n* = 11 case, in that it yields a partitioning of the chips that is markedly different, both visually and as determined by various more objective measures, from the partitioning of the space by natural languages with eleven color terms. But, as will be seen in the general discussion, the comparisons do cast aspersions on what otherwise might look like a promising explanation of the discrepancy between RKK’s results for the *n* = 6 case, replicated in this paper, and the new results for the *n* = 11 case.

## 4 General discussion

RKK claim that universal color naming reflects an optimal partitioning of color space. A reasonable expectation would therefore be for RKK’s model to predict the partitioning of color space by the eleven color universals, not just the first six of the BCTT evolutionary sequence. But our application of RKK’s model to the case of eleven color universals did not yield a partitioning in line with that expectation. Thus, the results reported in the previous section appear to imply that RKK’s model does not account for universal categorization, at least not fully. We consider four possible responses to this claim.

First, some might want to argue that the categories brown, pink, purple, orange, and gray are not predicted by RKK’s model simply because these categories are *not* universal; only the first six categories of the BCTT sequence (white, black, red, yellow, green, and blue) are. However, this response ignores the claims put forward by Berlin and Kay [9], according to which there are eleven universals, a subset of which is instantiated in a given language at a given time. Based on this proposal they are able to contend that lexicons evolve, so as to gradually include more universals. Thus, lexicons at the same evolutionary stage partition the space in the same way regardless of how different these languages are. And lexicons that partition the space differently are conjectured to be at different evolutionary stages. This is the heart of the BCTT’s response to cultural relativism. Also, and importantly, objecting that there only are six universals would redefine the notion of universality in a way that jeopardizes the BCTT. Color categories are considered to be universal on the basis of being observed across languages. Terms equivalent to the English “brown,” “pink,” “orange,” “purple,” and “gray” have been observed in almost all languages of industrialized societies, as have terms equivalent to “white,” “black,” “red,” “yellow,” “green,” and “blue.” (See Berlin and Kay [9], and the WCS data, at http://www1.icsi.berkeley.edu/wcs/; for a more recent contribution, see [25].) In short, it is problematic for proponents of universalism in color categorization to argue that only the first six categories are universal because that would imply that cross-cultural observation is not sufficient grounds to claim that there are universal patterns of color categorization.

Second, it could be said that, rather than showing the limitations of the hypothesis that universal color categories optimally partition color space, our results reveal a number of methodological concerns. We might have shown that Jameson and D’Andrade’s idea of a most informative categorization is not well captured by the procedure of optimizing *W* and requires a different function to be optimized. This is a possibility we cannot exclude, even though we *can* report here that applying any of the standard (e.g., PAM) and also not so standard (e.g., cross-entropy clustering from [48]) clustering algorithms to the *n* = 11 case did not improve on the PSO result; we also tried some variants of *W*, for instance, by weighing the *S* and *D* components of the function differently, again to no avail. It could also be argued that the failure of RKK’s model to accurately predict the categories in the *n* = 11 case results from an inadequacy of the stimuli. Specifically, finer-grained optimal partitionings might be hindered by the coarseness of the 330 color samples. Whether the use of the 330 Munsell chips from the WCS biased the clustering results is certainly a legitimate concern, for both the current study as well as RKK’s, and the same might be said for the assumption of CIELAB space (for various specific concerns about the stimuli or CIELAB space see, for instance, [17, 49, 50]).

Even granting that the foregoing are critical methodological concerns, however, the meaningfulness of the results of RKK’s implementation of Jameson and D’Andrade’s notion of most informative partitioning and its application to the 330 Munsell chips in CIELAB space cannot be ignored. After all, the model does have strong empirical backing from the results obtained for categorizations of up to six categories. It was therefore critical to explore the limits of the model with respect to its ability to explain the data concerning color categorization going beyond six categories. What we questioned is not RKK’s model, or the underlying methodological choices, but rather the model’s capacity to account for universal tendencies in color naming, granting that there are eleven, not six color universals. To that end, we applied RKK’s model to the case of eleven categories, using their stimuli—the 330 WCS chips—and also taking on board CIELAB space. What we found is that, even if we go along with all of RKK’s assumptions, their model fails to support the conclusion that optimal partitioning, as understood in terms of *W*-maximization, accounts for the emergence of universal color categories.

Third, it might be thought that Jameson and D’Andrade’s [18] already implicitly contains an explanation for the negative main result of our paper when they suggest that their informativeness criterion may yield less clear-cut predictions for finer-grained partitionings. Specifically, they claim that after the *n* = 3 partitioning into light/warm (WHITE), dark/cool (BLACK), and RED, “it becomes more difficult to determine which is the next most distant region” (p. 312). That optimizing *W* works better for lower *n*, with predictive accuracy gradually decreasing as the value of *n* increases, is a hypothesis worth exploring more systematically, and also one we flag here as an avenue for further research. We do want to note, however, that the results from our own simulations seem to somewhat militate against this explanation. After all, if *W* worked better for lower *n*, then one would expect to get a more determinate prediction in the case of *n* = 3 than in the case of *n* = 6. Instead, we obtained for the *n* = 3 case rather differently-looking clusterings all approaching the maximum *W*-value, something that did not occur in the *n* = 6 case. So, contrary to what one would expect if Jameson and D’Andrade’s suggestion were correct, the *n* = 3 case appears to be more indeterminate than the *n* = 6 case.

This brings us to a fourth possible answer of why optimizing *W* succeeds in the *n* = 6 case but fails in the *n* = 11. Since Hering [51], it is generally held that white, black, red, green, yellow, and blue have a special status in our color experience (see also [52]). However, *why* these colors are special is still an open question. While Hering’s “unique hues” (as the aforementioned colors are commonly called) were initially arrived at by introspection, toward the middle of the 20th century they came to be (tentatively) identified with neuronal responses in our processing system ([53]; see also [11, 12]). However, this hypothesis was questioned in later research [54] and ultimately got rejected [55–57]. That optimizing *W* for *n* = 6 produces precisely Hering’s unique hues while applying the same procedure to the *n* = 11 case does not produce the eleven universal color categories suggests that Hering’s hues are optimal, rather than the eleven universal categories.

However, this explanation is not supported by the results concerning the *n* = 7 to *n* = 9 cases presented at the end of the previous section. When comparing the number of random clusterings that were better represented by the PSO solutions for those cases than were the relevant WCS languages, we found that for *n* ∈ {7, 8, 9}, the results were not very different from those obtained for the *n* = 6 case. For the *n* = 10 and *n* = 11 cases, there were then suddenly many more “better represented” random clusterings.

We do not have a *specific* explanation for our negative main result, but, taking our cue from Liljencrants and Lindblom’s [32] work on vowel systems (as RKK did before us), we do want to point to a possible *general* explanation. It is long known that, although the human vocal tract can produce an indefinite number of vowels, only few of those are actually to be found in spoken languages [58]. Liljencrants and Lindblom try to explain this in terms of a principle of acoustic contrast optimization, according to which vowels are so chosen that the total distance between pairs of them, as measured in vowel space, is maximal. To test their hypothesis, Liljencrants and Lindblom built a computer program which calculates the coordinates in vowel space of n maximally contrastive vowels, for *n* ∈ {3,…,12}. The outcomes they obtain are nearly perfect for the *n* = 3 to *n* = 6 cases: for those cases, the computational models predict accurately the vowels found in spoken languages with the corresponding numbers of vowels. The computational results for larger vowel systems are not as good. Liljencrants and Lindblom conclude from this that, while the principle of acoustic contrast optimization is an important part of the answer to which vowels are selected for use in a language, there are likely to be further factors involved in the selection process, factors not implemented in their model. They conjecture that articulatory considerations, in particular ease of articulation and co-articulability (p. 854), are among such factors.

It is reasonable to believe that something similar applies to RKK’s model, and that while maximizing well-formedness is an important organizing principle in categorization, it is not the only such principle. As in Liljencrants and Lindblom’s model, the absence, in RKK’s model, of further organizing principles may make itself felt only if we transcend some level of complexity. In light of results which provide evidence for the role of social interaction in color categorization [16], [59], or those which provide evidence for the role of the distribution of color in the environment [15], we conjecture that the commonalities in the color lexicons of languages with greater numbers of color terms are partly to be explained in broadly cultural terms, for instance, as resulting from interactions between different language communities with shared interests, or also from languages having common ancestors [60], or again from the interaction between cultural needs and environmental factors [61]. Further exploration of this hypothesis must be left to another occasion.

## Appendix

To carry out the hydroPSO optimization procedure that we used for our research, one needs the CIELAB coordinates of the Munsell chips shown in Fig 1. These can be downloaded from the WCS website at http://www1.icsi.berkeley.edu. One further needs to code RKK’s well-formedness function *W* in R, which is a straightforward task and which we leave to the reader. Then the package allows one to code the actual optimization procedure in a highly compact manner (this package needs to be loaded together with the parallel package). Specifically, we used for this the following function, which takes a number *n* as input and outputs a clustering with *n* clusters:

psoClust <- function(n) {

 pso <- hydroPSO(startValues, W,

  lower = c(rep(min(lab$V1), n),

   rep(min(lab$V2), n),

   rep(min(lab$V3), n)),

  upper = c(rep(max(lab$V1), n),

   rep(max(lab$V2), n),

   rep(max(lab$V3), n)),

  control = list(MinMax = 'max',

   parallel = 'parallel'))

 prm <- matrix(pso$par, n)

 dst <- apply(prm, 1, rkkDist)

 out <- apply(dst, 1, which.min)

 return(out)

}

Here, startValues is a set of n CIELAB coordinate triples that the procedure is to take as a starting point, *W* refers to the definition of RKK’s function *W*, lab is the data frame containing the CIELAB coordinate triples of the Munsell chips, and rkkDist calculates the CIELAB distance from the *n* coordinate triples in prm to each of the coordinate triples in lab (the coding is again straightforward). The output is a vector of length 330, with element *i* assigning a label *l*_*i*_ ∈ {1,…,*n*} to the Munsell chip whose coordinates are given by the *i*-th row of lab.

To obtain a value for startValues, we generated 100 sets of *n* CIELAB coordinate triples and then picked the one with the highest *W*-value. Different approaches are possible. For the *n* = 11 case, we also used as starting position the eleven medoids that were obtained by applying the function in R to lab (asking for eleven clusters), and following a suggestion by Alejandro Parraga, we further used the eleven focal points of the mode map for French shown in Fig 6. Neither led to an improvement in the clustering output, whether visually or in terms of the cluster comparison indices used in Section 3.
